# Making progress

**DOI:** 10.7554/eLife.97430

**Published:** 2024-04-17

**Authors:** Jurgen Denecke

**Affiliations:** 1 Centre for Plant Sciences, School of Biology, Faculty of Biological Sciences, University of Leeds Leeds United Kingdom

**Keywords:** Golgi apparatus, super-resolution live imaging, membrane traffic, cisternal maturation, ERGIC, endoplasmic reticulum-Golgi intermediate compartment, GECCO, Golgi entry core compartment, *S. cerevisiae*

## Abstract

Mapping proteins in and associated with the Golgi apparatus reveals how this cellular compartment emerges in budding yeast and progresses over time.

**Related research article** Tojima T, Suda Y, Jin N, Kurokawa K, Nakano A. 2024. Spatiotemporal dissection of the Golgi apparatus and the ER-Golgi intermediate compartment in budding yeast. *eLife* **13**:e92900. doi: 10.7554/eLife.92900.

All eukaryotic cells have an endomembrane pathway which acts like an internal plumbing system that traffics biochemical compounds to various destinations inside the cell. At the centre of this network is the Golgi apparatus, which receives newly synthesised proteins from the endoplasmic reticulum at its *cis* end, and then sorts them to various parts of the cell.

The Golgi apparatus is made up of flattened membrane-bound sacs called cisternae which each gradually mature from being a *cis* compartment to being a *trans* compartment. Despite their conserved role, the way cisternae are organised is morphologically diverse (Figure 1A). In vertebrates, they are arranged into polarised stacks (with *cis* cisternae on one side and *trans* cisternae on the other), which are joined together into a tight ribbon (Klumperman, 2011); whereas in plants and insects, the stacks form individual bodies or mini-stacks dispersed throughout the cell. The cisternae of budding yeast, however, are not stacked together but physically separated (Nakano, 2022). This unique structure makes budding yeast a useful model system for studying how the *cis* Golgi matures into *trans* cisternae (Figure 1; Losev et al., 2006; Matsuura-Tokita et al., 2006). Now, in eLife, Takuro Tojima and co-workers from the RIKEN Center for Advanced Photonics and University of Tsukuba report a fascinating temporal roadmap of how this cisternal progression occurs (Tojima et al., 2024).

**Figure 1.**
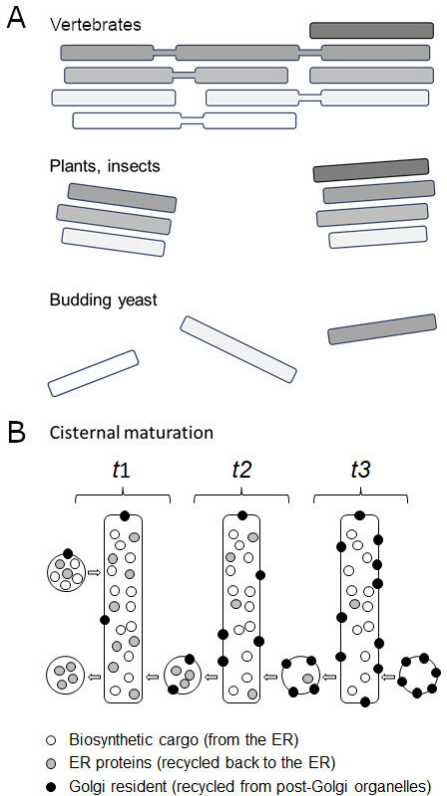
Golgi morphologies and the complexity of cisternal maturation. (**A**) Vertebrates (top) contain a large cluster of polarized interconnected stacks, made up of *cis* (white), *medial* (light grey) and *trans* (darker grey) cisternae. Plants and insects (middle) show similar stacks, but they are independent from each other. Budding yeast (bottom) do not contain Golgi stacks: instead *cis*, *medial* and *trans* cisterna are separate organelles, making them a useful model system for studying cisternal progression. (**B**) Illustration shows cisternal maturation of an early Golgi compartment with each rectangle representing the same cisterna at a different time point (t). Over time, the composition of molecules in the cisterna gradually changes due to certain proteins (grey circles) being recycled back via transport carriers towards the endoplasmic reticulum (retrograde transport, from right to left). The cisterna also receives biosynthetic cargoes (white circles) from the endoplasmic reticulum (ER) which pass through the Golgi to either be secreted at the plasma membrane or to reside in a vacuole. Proteins destined for the Golgi (black circles) are recycled back from later cisternae and post-Golgi organelles (not shown in the illustration) causing them to accumulate within the cisterna. This efflux and influx of carriers containing various proteins maintains the Golgi membrane and causes the concentration of molecules in the cisterna to decrease, increase or remain the same over time. For simplicity, the illustration does not include (i) peripheral Golgi-associated proteins, (ii) later *trans* cisternae that segregate proteins for the vacuole from secreted proteins, and (iii) retrieval of cargoes from post-Golgi organelles. In addition, transport carriers are symbolized as circles but can either be vesicles or tubules.

Cisternal progression was initially viewed as a result of selected proteins and membranes recycling back towards the endoplasmic reticulum. But the field soon realised that this cannot explain how the membrane surfaces of cisternae are maintained, nor the large portfolio of Golgi resident proteins that accumulate during cisternal progression. Therefore, Golgi cisternae are now thought to mature as a result of the influx and efflux of molecules (Figure 1B). In addition, the Golgi apparatus receives membranes and proteins not only at the *cis* end, but also at its *trans* compartments from post-Golgi organelles. Likewise, both ends of the Golgi actively export membranes and proteins too. This highlights the need to monitor Golgi maturation alongside other elements of the endomembrane pathway in the context of living cells.

Tojima et al. studied cisternal progression in live budding yeast cells using their newly developed microscopy technique which can simultaneously image two fluorescent proteins over time at super resolution (Kurokawa et al., 2019; Tojima et al., 2023). This enormous technical advance allowed the team to map how pairs of proteins involved in the endomembrane pathway spatially changed over time during cisternal progression. Such a vast portfolio of Golgi-resident markers, as well as temporary Golgi-associated proteins, had never been studied before. This led to the discovery of cisternal microdomains that had not been previously observed.

How and where *cis* cisternae initially emerge in yeast cells is also poorly understood. The minimalistic or reductionist view is that they form de novo when export vesicles from the endoplasmic reticulum fuse with each other (Glick, 2002). Alternatively, it has been suggested that a new Golgi apparatus can only be formed by making a copy of an existing one (Pelletier et al., 2002). The findings of Tojima et al. suggest that early Golgi cisternae are not simply made from vesicles fusing together. Instead, a structure called the Golgi entry core compartment (GECCO), which had previously only been observed in plants (Ito et al., 2018), is required to accept cargoes from the endoplasmic reticulum, suggesting it goes on to form the *cis* cisternae in budding yeast.

Another highlight of this study is the surprising behaviour of Ypt1, the yeast version of the Rab1 enzyme in mammalian cells. Tojima et al. found that Ypt1 appears at the earliest Golgi cisternae and then again much later at the *trans*-Golgi network, the final part of the Golgi which transports cargo proteins to vacuoles and the plasma membrane. Nature tends to re-purpose successful mechanisms. The findings of Tojima et al. suggest that Ypt1 is needed to deal with not only cargoes arriving and exiting at the *cis* Golgi, but also those being received and exported at the *trans* end.

Unlike *cis* cisternae, which only interface with a single organelle (the endoplasmic reticulum), *trans* cisternae have to control transport to vacuoles and the plasma membrane. But to maintain homeostasis, there are equally important recycling routes from these two locations back to the Golgi apparatus. In this respect, it still remains to be seen if all traffic proceeds via the *trans*-Golgi network, or if secreted and vacuolar proteins exit the Golgi apparatus at different subdomains.

In addition, the study by Tojima et al. only focused on Golgi-resident and Golgi-associated proteins, and many other elements of cisternal progression and the endomembrane pathway remain to be explored. For instance, the microscopy technique applied in this study could be used to follow post-Golgi organelles and how they mature. Cargo proteins are also notoriously difficult to detect at the Golgi apparatus due to being transported to their end location very efficiently. Increasing the sensitivity of high-speed fluorescent microscopy may allow researchers to track individual secreted and vacuolar proteins more easily as they progress through and exit from the Golgi. It seems that there are many more players to add to this exciting roadmap of cisternal progression.
